# Improving voltage-sensitive dye imaging: with a little help from computational approaches

**DOI:** 10.1117/1.NPh.4.3.031215

**Published:** 2017-05-19

**Authors:** Sandrine Chemla, Lyle Muller, Alexandre Reynaud, Sylvain Takerkart, Alain Destexhe, Frédéric Chavane

**Affiliations:** a Aix-Marseille Université, Centre National de la Recherche Scientifique (CNRS), UMR-7289 Institut de Neurosciences de la Timone, Marseille, France; b Salk Institute for Biological Studies, Computational Neurobiology Laboratory, La Jolla, California, United States; c McGill University, McGill Vision Research, Department of Ophthalmology, Montreal, Quebec, Canada; d Unit for Neurosciences, Information and Complexity (UNIC), Centre National de la Recherche Scientifique (CNRS), UPR-3293, Gif-sur-Yvette, France; e The European Institute for Theoretical Neuroscience (EITN), Paris, France

**Keywords:** voltage-sensitive dye imaging, computational models, biophysical model, advanced signal processing

## Abstract

Voltage-sensitive dye imaging (VSDI) is a key neurophysiological recording tool because it reaches brain scales that remain inaccessible to other techniques. The development of this technique from *in vitro* to the behaving nonhuman primate has only been made possible thanks to the long-lasting, visionary work of Amiram Grinvald. This work has opened new scientific perspectives to the great benefit to the neuroscience community. However, this unprecedented technique remains largely under-utilized, and many future possibilities await for VSDI to reveal new functional operations. One reason why this tool has not been used extensively is the inherent complexity of the signal. For instance, the signal reflects mainly the subthreshold neuronal population response and is not linked to spiking activity in a straightforward manner. Second, VSDI gives access to intracortical recurrent dynamics that are intrinsically complex and therefore nontrivial to process. Computational approaches are thus necessary to promote our understanding and optimal use of this powerful technique. Here, we review such approaches, from computational models to dissect the mechanisms and origin of the recorded signal, to advanced signal processing methods to unravel new neuronal interactions at mesoscopic scale. Only a stronger development of interdisciplinary approaches can bridge micro- to macroscales.

## 1 Introduction

**Fig. 1:**
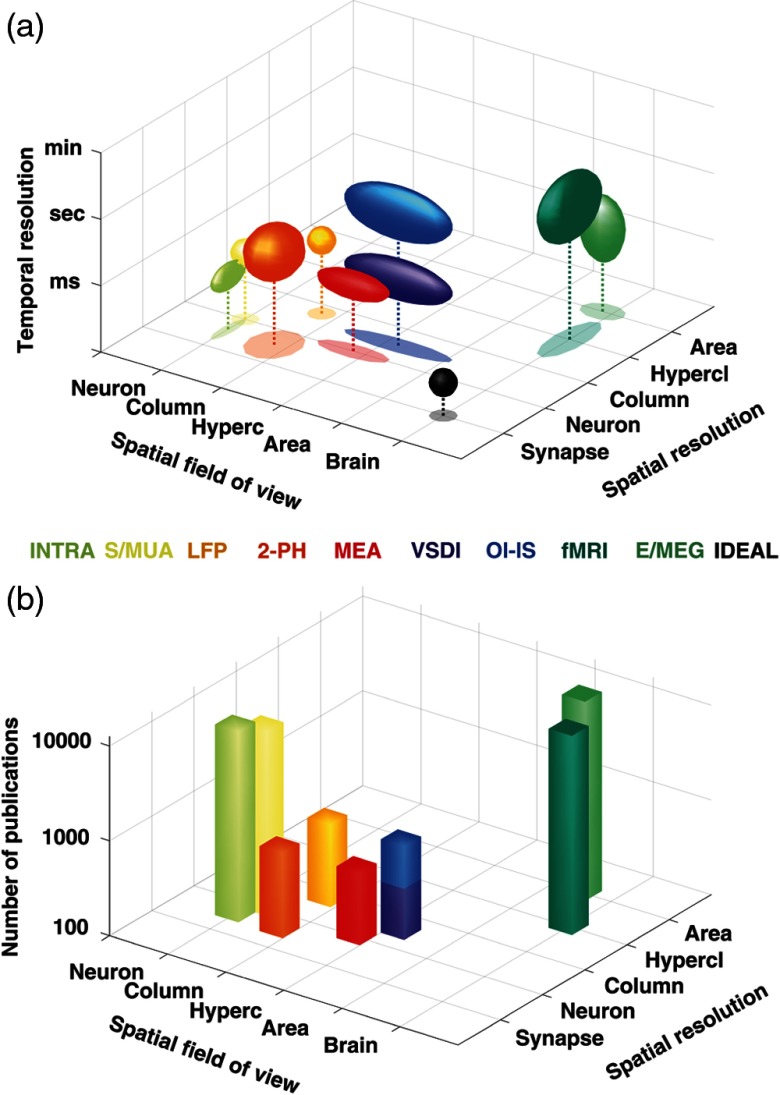
Spatio-temporal resolution and scales of neuronal recording methods. (a) Three-dimensional representation of 10 families of neuronal recording methods as a function of their spatial resolution, temporal resolution, and the spatial field-of-view that they can reach.^2^ INTRA, intracellular recordings; S/MUA, single or multiunit activity; LFP, local-field potentials; 2-PH, two-photon microscopy; MEA: multielectrode array; VSDI, voltage-sensitive dye imaging; OI-IS, optical imaging of intrinsic signals; fMRI, functional magnetic resonance imaging; E/MEG, electro- or magneto-encephalogram; IDEAL, the ideal technique. (b) In the same spatial resolution, field-of-view representation, frequency histograms of the amount of publication referenced in PubMed with the word “cortex” and one of these method. The generic search line was: (TECHNIQUE_NAME[Title/Abstract]) AND (cortex[Title/Abstract] OR cortical[Title/Abstract]). When appropriate, the technique name was written in full and abbreviated.

Despite the tremendous recent advancements in neuronal activity recording tools, voltage-sensitive dye imaging (VSDI) remains the only technique that allows to measure neuronal activity with high temporal (1 to 10 ms) and spatial (<50  μm) resolution^1^^,^^2^ over a large field-of-view (typically about 1 to $2\;{\mathrm{cm}}^{2}$). VSDI^2^ thus provides access to the mesoscopic scale, i.e., a network of neurons from the column to a whole area, between microscopic (single-neuron) and macroscopic (whole-brain) scales. Recording techniques providing access to this scale—VSDI as well as by other optical imaging techniques (e.g., optical imaging of intrinsic signals, two-photon microscopy) and multielectrode arrays [Fig. 1(a)]—have unfortunately received relatively little attention from the neuroscientific community: only 4% of publications listed in PubMed with the keyword “cortex” study this mesoscopic scale, with only 0.5% from VSDI [Fig. 1(b) and Table 1]. This relative lack of attention stands in contrast to the fact that more than 95% of interneuronal connections occur between neurons separated by less than 2 mm in cortex.^4^ These under-explored techniques, with VSDI in particular because it remains unique in the field-of-view and temporal resolution it reaches, have, therefore, an enormous potential for unraveling new fundamental scientific discoveries. Two points, however, stand in the way of this potential: (1) insufficient understanding of the VSDI signal’s origin and (2) the lack of standardized signal processing tools. Developing dedicated computational approaches may, therefore, be the key to improve our knowledge and know how in VSDI studies.

Here, we review recent advances in that direction. First, we describe how generative models of VSDI data can help to better understand the origin of the signal and unravel possible underlying mechanisms. In particular, biophysical models have proved very useful in describing the signal’s multicomponent origin. Second, we strongly suggest a standardization of signal processing tools that are key for generalization and comparison of results obtained with VSDI. With this imaging technique, the raw signal is corrupted by many noise components arising from physiological, mechanical, or electronical sources. We review the signal processing tools that have been developed for VSDI signal analysis. Finally, we show that combining single-trial data analysis with computational models can reveal the origin of the observed signal’s dynamics. Similar to the convergent interdisciplinary efforts that have been undertaken to optimize our understanding and use macroscopic brain imaging signals [i.e., functional magnetic resonance imaging (fMRI), magneto-encephalogram, and electro-encephalogram], we believe that such computational approaches are needed for the development of VSDI and are vital to establishing this as a standard neuroscientific imaging technique.

**Table 1:** Orders of magnitude of neuronal recording techniques in terms of spatial resolution, temporal resolution, and field-of-view,^2^^,^^3^ as well as the exact hit in PubMed search.

	INTRA	S/MUA	LFP	2-PH	MEA	VSDI	OI-IS	fMRI/TEP	E/MEG	IDEAL
Spatial resolution	Subneuronal	Neuron	Neuron-column	subneuronal	Neuron	Neuron-column	Neuron-column	Subareal	Subareal	Subneuronal
Field-of-view	Neuron	Neuron	Column	Column	Multicolumns	Area	Area	Brain	Brain	Brain
Temporal resolution	Sub-ms	ms	Tens of ms	Tens of ms	ms	ms	Hundreds of ms	Hundreds of ms	Hundreds of ms	Sub-ms
Pubmed search:	10,267	8680	739	784	572	346	964	24,943	16,131	

## 2 Computational Models of Voltage-Sensitive Dye Imaging Signal

We start by reviewing the different computational models available to better understand the origin of the population dynamics and the VSDI signal.

### 2.1 Dissecting the Underlying Mechanisms of the Response Dynamics

The behavior of large assemblies of neurons can be studied without having to tackle the mathematically unwieldy challenges associated with microscopic considerations. Indeed, at the mesoscopic scale, it is valid to study average values, thus requiring only simple computations to describe the activity of interacting populations of neurons through mean-field theory.^5^ The VSDI signal reports mesoscopic population activity at high spatio-temporal resolution and has been successfully reproduced by models developed at this scale.^6^^–^^15^ Here, we briefly review, in a nonexhaustive manner, three families of such models (see Ref. 14 for a more detailed overview of the models and their equations).

#### 2.1.1 Neural-field models

Grimbert and Chavane,^10^ as well as Markounikau et al.^13^ and Deco and Roland,^14^ proposed neural fields as a suitable mesoscopic model of cortical areas. Neural fields are continuous networks of interacting neural masses, describing the dynamics of the cortical tissue at the population level.^16^ Therefore, they are appropriate to solve the direct problem of the VSDI signal, i.e., to generate a VSD signal given the neural substrate parameters and activities. These three models indeed account for physiological spatio-temporal dynamics of V1 population responses to various illusory motion stimuli (apparent motion, line motion).

#### 2.1.2 Self-organizing models

The laterally interconnected synergistically self-organizing map (LISSOM) family of models^17^^,^^18^ was also proposed to reproduce the spatial organization of V1 as observed with optical imaging. It is based on Hebbian self-organizing algorithms^19^ used to visualize and interpret large, high-dimensional data sets. Sit and Miikkulainen^9^ but also Stevens et al.^6^ proposed variants of the original LISSOM model to account for the development of stable and realistic cortical functional maps.

#### 2.1.3 Conductance-based models

Rangan et al.^8^ proposed a large-scale conductance-based integrate-and-fire (IAF) model of the primary visual cortex in order to reproduce the spatiotemporal activity patterns of V1, as revealed by VSDI, in response to the line motion illusion.^20^ This family of models simplifies the model of Hodgkin and Huxley (HH) by representing neurons as IAF units while still taking into account a simplified version of the conductance changes due to action potentials.^21^ More recently, Chizhov^15^ also used this family of models to account for VSDI dynamics.

Each of these models helps understanding or testing the role of some specific components of the VSDI signal. For instance, one intriguing feature of VSDI cortical responses to illusory motion stimuli,^20^^,^^22^ calls for the existence of slow mechanisms that can “bind” spatially and temporally the transient stationary inputs composing the stimulus sequence. In all models, such nonlinear low-pass filtering by the neuronal population has been attributed to various mechanisms. For instance, Markounikau et al.^13^ and Chemla and Chavane^23^ both suggested that a balance between excitation and inhibition and lateral connections are potential mechanisms shaping the sequence of stationary input. Similarly, Chizhov^15^ found that intracortical connectivity is a critical factor accounting for VSDI dynamics. In Ref. 8, the NMDA conductance has been further proposed as a complementary, nonexclusive, mechanism to account for the slow dynamics of the VSDI signals. Such a conductance could also play an important role in structuring on-going spontaneous activity by generating an intermittent unsuppressed state.^24^ These results show that computational models can be used to demonstrate the plausibility of various mechanisms probed by specifically incorporating the putative candidate, conductances, or connectivity—such as horizontal and vertical intercolumnar connections between neural masses^10^ or feedback.^14^ However, none was specifically designed to dissect the origin of the VSDI signal, which is a central question for interpreting the results (see Ref. 14 for a detailed review on this problematic). The VSDI technique is indeed a complicated signal based on voltage-sensitive dyes that bind to the cells’ membrane and linearly transform variations in the membrane potential into fluorescence. A millisecond temporal resolution is reached by using a highly sensitive charge-coupled device camera, whereas the spatial resolution (down to 20 to 50  μm) is mainly limited by optical scattering of the emitted fluorescence.^25^ The recorded signal, therefore, results from fluorescence integrated over a large population of cells and is thus affected by activity of intermingled components under each measuring pixel, e.g., different neuronal compartments (including dendrites, somata, and axons) of different cell types (excitatory and inhibitory) in different layers, which are likely to be stained in the same manner. How to isolate the contributions from its different components is, therefore, a difficult question to answer directly. To specifically investigate this question, Chemla and Chavane^23^ have proposed a biophysical model that we present below.

### 2.2 Biophysical Model for Unraveling the Signal’s Origin

#### 2.2.1 Basics of the model

**Fig. 2:**
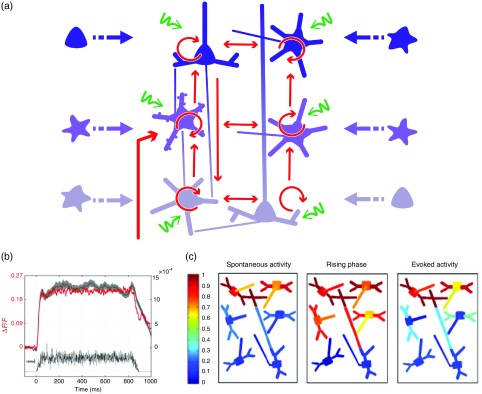
VSDI biophysical model schematic and contributions. (a) Model representation. The six populations of neurons, depicted by one unique representative neuron (small pyramidal cells in layer 2, spiny stellate cells in layer 4, large pyramidals in layer 5, and smooth stellate cells in each layer), are recurrently connected (red arrows). The cortical column is embedded into a larger network by simulating a realistic synaptic bombardment on each population (green arrows) and by modeling lateral connections between the column and its neighbors (blue dashed arrows). Inputs from the thalamus to layer 4 neurons are represented by the large red arrow on the left. (b) Time-course of the modeled VSDI signal (red trace) in response to a thalamic input of 800 ms (black trace), compared to the experimental signal (gray trace) obtained in monkey V1. (c) Correlation analysis between the VSDI signal and the membrane potential of each compartment of the column for three periods of time (spontaneous activity, stimulation onset, or rising phase and evoked activity). (Adapted with permission from Ref. 23. Copyright © 2010.)

We developed a detailed biophysical cortical column model based on known neural properties of the visual cortical network and adjusted to reproduce the dynamics of experimental VSDI signal.^26^^,^^27^ The model was developed at a scale that corresponds in size to one pixel of the VSDI image (50  μm) and embedded into a larger network to be realistic, i.e., an artificial hypercolumn (in the case of V1). More precisely, this model comprised 180 multicompartment HH neurons [see Fig. 2(a)] with three different types of excitatory neurons (one type per represented layer) and one unique type of inhibitory neurons in each of the three layers (2/3, 5, 5/6). Excitatory and inhibitory neurons, which represent 80% and 20% of the cells respectively, were initially fitted to intracellular recordings from Ref. 28. These neurons were then recurrently connected in accordance with Ref. 29, which provided a quantitative estimation of the synaptic projections between these different neuronal types. The local network calibration was done by tuning the contrast response functions of these two populations of neurons to reproduce those obtained electrophysiologically *in vivo*.^30^ Lateral interactions were tuned in strength and number of synapses to fit excitatory and inhibitory distributions, respectively, from Refs. 31 and 32. Background activity was also taken into account in the form of fluctuating ionic conductances^33^ in order to reproduce *in vivo* synaptic bombardment. Finally, the dye attenuation parameter was given by the distribution of fluorescence intensity estimated by Ref. 34.

#### 2.2.2 Unraveling time and stimulus-dependent origin of the signal

We computed the VSDI signal by linearly integrating the membrane potential over the total surface area, corrected by a factor accounting for the amount of staining of each compartment. This model reproduced well the experimental VSD signal dynamics [Fig. 2(b), in black the mean±SEM of the recorded VSDI and in red the modeled VSDI]. At this stage, the model was used to quantify the different contributions of the signal, by computing the fraction of the contribution of each compartments, but also the correlation between each of these compartment and the global signal [represented graphically in Fig. 2(c)]. Importantly, the model was very stable and tolerant to changes in the model’s parameters (synaptic weights and number of connections).

As expected, the VSDI signal mainly reflects dendritic activity of excitatory neurons in superficial layers [gross contribution of 60%, high level of correlation in Fig. 2(c)], with 40% of the signal originating from a mixed contribution of inhibitory neurons, lower layers, and axons (hence spikes). However, when increasing the thalamic input strength, these contributions are changing, inhibitory cells contribution increase, and the one of axons decreases. Importantly, these contributions are also dynamic: the contribution and level of correlation from layer 4 neurons and inhibitory neurons, as well as axons, increased transiently at response onset [Fig. 2(c)].^23^ This model hence demonstrated that the relative contribution of all compartments is not stationary and that no compartment on his own allows to fully account for the global signal.

#### 2.2.3 Effect of anesthesia and the notch as a marker of transient imbalance of excitation/inhibition

**Fig. 3:**
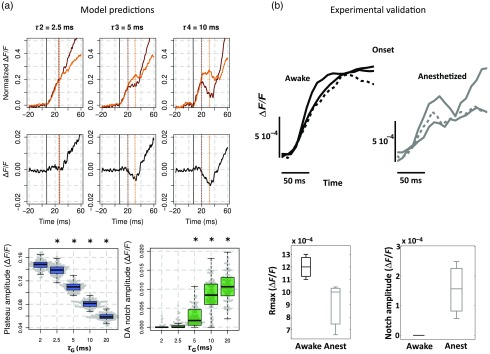
Model predictions on the effect of anesthesia and experimental validation. (a) Effects of tauG modulation on the modeled VSDI signal dynamics (Plateau and DA notch amplitude). Top row: onset of the normalized VSDI signal time-courses decomposed into excitatory (burgundy traces) and inhibitory (orange traces) cells activity for three tauG values (2.5, 5, and 10 ms), revealing the DA notch formation. Middle row: time-course of the difference between excitatory and inhibitory VSDI signals. Bottom row: boxplot diagrams of the plateau amplitude (left) and the DA notch amplitude (right) of the modeled VSD response as a function of tauG. Significant differences with the condition tauG=2  ms (P<0.01) are denoted by a star. (b) Experimental validation of the model predictions shown in (a). Top row: onset of the experimental VSDI signal time-courses obtained in three awake (left) and three anesthetized (right) monkeys, in response to full-field drifting gratings of high contrasts. One monkey was recorded in both arousal conditions (dashed lines). Bottom row: Boxplot diagrams of the Rmax or plateau amplitude (left) and DA notch amplitude (right) values of the experimental VSDI data shown on top. (Adapted with permission from Ref. 35. Copyright © 2016.)

In a recent extension of this model, we further probed how much these contributions will depend on the network state since studies in VSDI are both done in anesthetized and awake preparations.^35^ Succinctly, we manipulated a key model parameter to account for the effect of anesthesia: the decay time constant tauG of GABA_A_-mediated IPSCs. Indeed, tauG has been shown to be prolonged in a dose-dependent manner by most anesthetics as reported in Ref. 27. Figure 3(a) shows that, when increasing tauG, our model predicted that the VSDI signal amplitude should decrease and the transient imbalance between excitation and inhibition increase. These predictions were confirmed experimentally with monkeys at different arousal states, induced by the administration of midazolam, i.e., a positive GABA_A_ modulator, during a behaving session [Fig. 3(b)]. Altogether, these results provide a quantitative description of the effect of GABA_A_ receptor modulation on the cortical population dynamics, as measured by the VSDI signal. Interestingly, our model predicted that one key feature of the VSDI signal, the so-called “deceleration–acceleration (DA) notch” component introduced by Sharon and Grinvald^36^ as “a small transient drop in the rate in which the evoked response increased,” is resulting from desynchronization between excitation and inhibition induced by anesthesia. Physiological recordings in awake and anesthetized preparation confirmed this prediction. The DA notch was indeed proposed to be an emergent signature of the cortical network excitability.^36^^–^^38^ Chemla and Chavane’s study further demonstrated that this is a prominent property of VSDI in anesthetized state and could be taken as a marker of a transient imbalance between excitation and inhibition. This effect is logical since anesthetic specifically slows down inhibitory conductances and not excitatory. At response onset, inhibition will have the possibility to transiently override excitation, before being re-equilibrated by the strongly recurrent cortical network.

VSDI generates complex experimental data that are difficult to interpret. Computational models can help explain the origin of this signal. These models are useful to understand the role of the major components of the VSDI signal and to generate experimentally testable predictions. However, the validation of such models cannot be achieved in isolation to real experiments, which are necessary to constrain them. In order to facilitate the confrontation of computational models and real data, it is, therefore, necessary to improve the signal processing algorithms available for VSDI data, and we now review the recent literature dedicated to this.

## 3 Signal Processing Methods for Improving Voltage-Sensitive Dye Imaging Signals

### 3.1 Review of the Different Methods

The raw VSDI signal is a noisy combination of several different components. We describe below the three main families of denoising methods that have been proposed to extract the neuronal signature from VSDI data.

#### 3.1.1 Blank subtraction

The most common and first empirical denoising method is the blank subtraction.^1^^,^^39^ It consists in estimating noise components using blank trial recordings (no stimulation) or a “cocktail blank” consisting of the mixture of all other stimulation conditions.^40^ The first step of the analysis consists of dividing each image of the stack by the first frames (recorded before stimulus onset) in order to correct for inhomogeneous levels of illumination and staining. In the second step, image stacks collected during stimulated trials are subtracted by those acquired during blank trials on a frame-by-frame basis. This subtraction aims at removing the slow drifts due to dye bleaching as well as synchronous physiological artefacts like heartbeat.^1^ A decaying general trend often persists, however, which can be attributed to change in bleaching dynamics or physiological parameters. Therefore, a subsequent linear detrending step can be applied.^41^^,^^42^ Some more statistically reliable measure can then be applied, such as a z-score taking into account the level of variability pixel by pixel.^20^^,^^43^

However, this blank subtraction method presents several limitations. The most important is that the first frames division is an inaccurate normalization method^44^ and leads to a systematic misestimation of the intertrial variance dynamics.

#### 3.1.2 Blind sources separation techniques

The first decomposition technique applied in intrinsic optical imaging was principal components analysis.^45^^,^^46^ The signal is decomposed using an automatic algorithm but signal versus noise modes need to be identified *a posteriori*, often using *ad hoc* statistical criteria. This was later improved by using multitaper harmonic analysis.^47^ However, noise sources synchronized on acquisition or stimulus onset can remain embedded in selected modes. Further improvements have been proposed, such as extended spatial decorrelation,^48^^,^^49^ which uses spatial statistical features to separate the recorded mixed sources, or indicator functions,^50^^–^^52^ which are determined upon stimulated/reference trials comparison.

The second main decomposition processing family is independent components analysis (ICA),^53^ which relies on the extraction of the original sources by maximizing their statistical independence. ICA has been mostly applied on VSDI recordings in anesthetized preparations at the single-trial level. *A posteriori* component identification also mostly relies on statistical criteria^54^^,^^55^ or with a “weak model” on intrinsic optical imaging data.^56^ Again, these decomposition techniques focus on the temporal dimension and can easily be combined with complementary spatial routines such as spatial ICA^57^ or local similarity minimization.^58^ As noted above, the modes are not determined upon physiological criteria but upon signal statistics. Thus, nothing guarantees perfect source separation and parameters estimation is impossible.^59^ Signal and noise modes can also be classified as user judgment after decomposition, for instance based on complementary recordings.^60^

These techniques can also be successfully applied on datasets obtained with specifically designed paradigms with periodic stimuli. There, the effective separation of the stimulus-evoked responses from noise is done with Fourier analysis, first developed for fMRI mapping^61^ and then applied to intrinsic^62^ and voltage-sensitive dye imaging.^38^^,^^59^^,^^63^

#### 3.1.3 Linear regression techniques

A different solution lies in multiple linear regression techniques, which were initially developed for fMRI^64^ and were subsequently adapted to various optical imaging techniques: intrinsic imaging,^56^^,^^65^^,^^66^ blood flow,^67^ calcium fluorescence,^68^ synaptoPhluorin fluorescence,^66^ and VSDI.^69^ These techniques are based on explicit decomposition of all signal components, mostly by identification of their physical sources. These components are then used to build a regression matrix on which the signal will be projected. The shape of the regressors, therefore, has to be modeled *a priori*. By construction, this technique has several advantages—it is applied at single-trial level, can specifically identify nonreproducible artefacts, and discounts for any bias in component selection. As with the other techniques, it can also be further constrained by the spatial structure of the signal.^70^

### 3.2 GLM for Recovering Single Trials

One key issue in linear regression techniques is the definition of components. They can be defined as templates or mathematical models. Templates can be used to model components that are very reproducible across trials and do not present any other changes than gain amplitude. Several studies have successfully used templates for modeling response components in intrinsic, synaptophluorine,^66^ or calcium imaging.^68^ However, they do not allow to account for changes in latency, duration, or shape, as seen in VSDI. One solution is to manually decompose the expected response in several template components,^65^^,^^67^ but this is only possible when changes in response shape are small, as in intrinsic imaging.

To overcome these technical limitations, important improvements come from the use of fMRI’s linear optimal basis sets (FLOBS) initially designed for fMRI^71^^,^^72^ in order to extract the activity dynamics in the VSDI signal and to model several evoked response shapes.^69^^,^^70^^,^^73^ A set of temporal regressors is determined by a singular value decomposition of a large set of simulated possible changes in the response. Then, few first eigenvectors are kept and included as regressors in the model. Such model is then able to selectively extract, independently for each pixel and each trial, a large range of temporal dynamics of responses evoked by different sets of stimuli, including changes in amplitude, duration, and delay. It should be noted, though, that in linear regression, good source separation requires that all the vectors of the new basis are orthogonal. Thus, when building the regression matrix, one must check that the nonneural components are orthogonal to the stimulus-evoked response ones in order to ensure that the evoked response signal is not embedded in noise regressors and that sensory-driven response estimated by the model is not corrupted by the noise sources. This is usually trivial for VSDI data as noise components often show a much faster dynamic and periodicity than response ones.

**Fig. 4:**
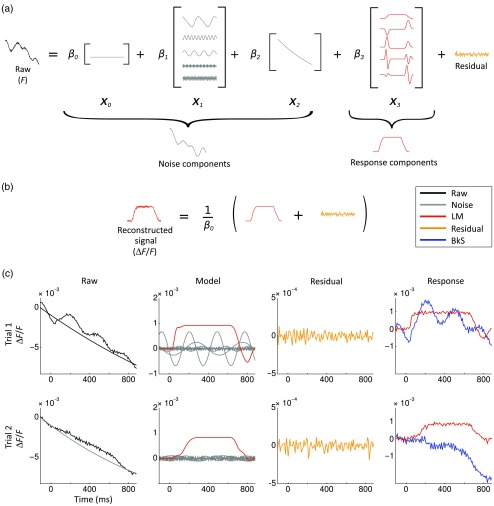
Linear model decomposition. (a) A raw trial is linearly decomposed into noise components ($X_{0}$ baseline, $X_{1}$ periodic components, and $X_{2}$ bleaching components), the evoked response components ($X_{3}$), and the residual. F denotes fluorescence. (b) Linear model denoising scheme. The reconstructed signal is the sum of the response components ($X_{3}$) and the residual, divided by the baseline illumination level to obtain a normalized reconstructed signal (ΔF/F). (c) Example of the linear model application on two trials in response to a-600 ms visual stimulation in the monkey visual cortex. First column: raw trials (black) and bleaching component (as estimated with the linear model; gray). Second column: other components estimated with the LM: evoked response (red) and periodic noise components (gray). Third column: residuals. Last column: estimated responses using the linear model denoising scheme (LM; red) and the standard blank subtraction (BkS; blue). Note the different scales on the ordinates axis. Adapted with permission from Ref. 69.

Figure 4(a) shows the model design used by Reynaud et al.^69^ to denoise VSDI data acquired in the awake monkey. The noise and response components are modeled in the following steps. The first noise component represents the baseline level $X_{0}$ by a constant value. Electronic^74^ and physiological artifacts $X_{1}$ (Ref. 1) are characterized by periodic oscillations and are thus modeled with Fourier series, allowing for phase changes. Finally, the dye bleaching can be defined with exponential functions $X_{2}$.^75^ The response components $X_{3}$ are the FLOBS and describe the neuronal response. The decomposition of the temporal signal on the basis of all these regressors will lead to the identification of individual coefficients. Finally, the response components and the residual can be extracted and normalized by $X_{0}$ to reconstruct the denoised response [Fig. 4(b)].

The application of the linear model to two experimental trials in response to a stimulation in the monkey visual cortex is shown in Fig. 4(c). The raw time courses are represented in black in the first column. For each raw signal, the noise components as identified by the model are shown in gray (first column for the bleaching and second column for the periodic components). The evoked response components are shown in red (second column). Bleaching, heartbeat, and fast oscillatory noises were clearly visible on these trials and were well captured by the model. The residuals (mostly white noise) are shown in the third column (orange). The signals, denoised with either the standard blank substraction (blue) or the linear model (red) are shown in the fourth column. As seen in these examples, the linear regression method provides a large improvement in signal to noise.^69^ Further improvement of the method by combining it to convex nonsmooth regularization priors has recently been proposed.^70^

This method thus recovers the signal at single-trial level and offers key data that more faithfully capture the variable population dynamics of cortical networks recorded in VSDI.^76^ Such variability can obsure important spatio-temporal features when averaging VSDI data. In particular, a key question is the question of the spatio-temporal response to small local stimuli. In awake nonhuman primates, spreads of activity have been shown^26^^,^^69^^,^^77^ but their origin and the question of whether they represent activity propagation within the cortical tissue have been disputed.^78^^–^^80^ Here, our denoising method has enabled us to conclusively answer this debate by applying a new wave detection approach to the data denoised for single-trial analysis. The combination of these LM-denoised data and further signal processing approaches has allowed us to demonstrate that traveling waves are at the origin of the spread emerging at the trial-averaged level.

### 3.3 Instantaneous Phase for Revealing Single-Trial Waves

Following denoising of the VSDI signal, further computational approaches are necessary to characterize the complex spatio-temporal dynamics of neocortical networks. In general, these methods aim to quantify spatio-temporal flow of activity in the optical imaging data, often with the goal to characterize propagation of spontaneous and stimulus-evoked waves, a difficult task in the context of the low signal-to-noise ratios commonly encountered when imaging awake behaving animals *in vivo*. For this task, various computational approaches have been introduced, including the space-frequency singular value decomposition,^81^^,^^82^ phase-gradient directionality,^83^ template matching,^84^ and an optic flow-based approach.^85^ In each case, these methods employ a mathematical representation of the optical imaging signal to derive a bounded measure of wave-like organization in the data, allowing to then estimate further quantitative characteristics such as propagation speed, wavelength, and direction. In order to conclusively test whether propagating waves are evoked in the primary visual cortex of the awake monkey in response to small visual stimuli, however, we adopted a statistical approach to wave detection using a phase-based measure.^86^

**Fig. 5:**
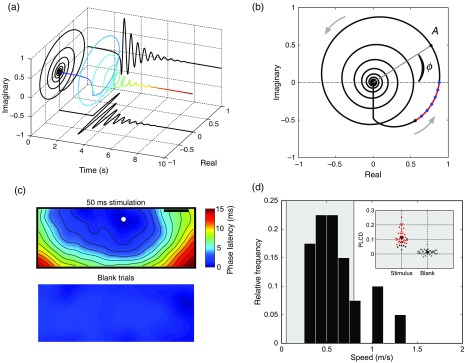
Phase latency method for detecting waves in single-trial data. (a) The real, imaginary, and complex plane projections of the analytic signal (line colored by heat in time) for a damped oscillation make explicit the decomposition of a real signal into a complex phasor. (b) The complex plane projection in the previous panel is used to analyze instantaneous amplitude (A, complex modulus) and phase (φ, complex angle) in the real signal. Gray arrows indicate the direction of phasor rotation in time. The black dot (bottom left) represents the starting point for the phase latency calculation. The blue dots represent discrete samples leading up to the phase crossing. (c) Average phase latency map for the region of interest in the primary visual cortex of awake monkey, in the stimulus (top) and black (bottom) condition. (d) Propagation speeds extracted from the slope of the relation of phase latency with distance in the unsmoothed maps, in the 50-ms stimulus condition. (Inset) Phase latency correlation with distance, stimulus (black) and blank (gray) conditions. Adapted with permission from Ref. 86.

We first employ the “analytic signal” representation^87^^,^^88^ to estimate phase at each pixel in the optical imaging data, as with previous approaches based on this mathematical framework.^83^ In this representation, a real-valued timeseries [such as values from one pixel of the imaging array; real plane, at bottom, Fig. 5(a)] is transformed into a complex-valued timeseries [line color-coded with increasing heat to indicate time, Fig. 5(a)]. In the complex plane projection [at left, Fig. 5(a)], the result is a “phasor” rotating in the complex plane, whose length (or modulus) represents signal instantaneous amplitude and whose angle (or argument) represents signal instantaneous phase. This estimate of signal phase at each time point can then be used to compare offsets in activity across many pixels, in a manner robust to noisy amplitude fluctuations.

Next, as a signal processing optimization, we introduce a measure termed “phase latency” [red curve, Fig. 5(b)], which quantifies the latency in absolute time to a given phase crossing in the complex plane. Specifically, by starting from a chosen point in the timeseries, for example, a point just before response onset [black dot, Fig. 5(b)], we can calculate the time of the next phase crossing at each pixel. This measure allows us to flexibly compare responses between pixels with slightly differing temporal frequencies, and in this way, it precludes the necessity to filter the data within a tight frequency range. This approach thus has the ultimate effect of reducing overall waveform distortion in the analyzed signal, an important consideration when working with bandpass filtered data. When the phase crossing of interest falls between two discrete samples [blue dots, Fig. 5(b)], the precise time for the crossing is calculated by linear interpolation between the two points based on their instantaneous frequency (dφ/dt).

With this measure calculated at each pixel, we then have a “phase latency” map starting from the chosen time point. We next quantify spatial patterns in these maps in several steps. First, we estimate the wave source from the minimum of the smoothed phase latency maps [depicted schematically with a white dot, Fig. 5(c)]. It is important to note here that if the phase latency map contains only noise, without spatiotemporal organization, then the estimated wave source will be at a random point determined by the noise fluctuations. Next, after calculating the Euclidean distance matrix from the source point, we can estimate propagation speed from the slope of the relation of phase latency with distance from the source [Fig. 5(d)] and the correlation coefficient quantifying the strength of this relationship ($\varrho_{d}$). We can then form a well-defined statistical test to determine whether significant phase organization exists in the VSDI data in each trial (one-tailed t-test, $H_{0}$: $\varrho_{d}=0$, $H_{1}$: $\varrho_{d}>0$, in our initial work). Thus, by testing phase organization systematically across all pixels in space, this approach maximizes one of the main strengths of VSDI—its high spatial resolution over a large field-of-view—to enable analysis at the single-trial level. The result of these calculations is a robust and sensitive approach for detecting arbitrarily shaped waves in very high-noise multichannel recordings, by means of a well-defined statistical test.

## 4 Discussion

In this review, we have provided a series of examples showing that computational approaches can standardize and generalize VSDI as an unmatched tool for studying instantaneous processing by neural circuits. The VSDI signal is indeed unique in the scales and resolutions it reaches (reviewed in Ref. 2) and has already allowed uncovering key dynamic processing and interactions within cortical networks, as shown in particular by Grinvald et al.^20^^,^^36^^,^^76^^,^^89^^–^^91^ The neuronal operations occurring at the mesoscopic scale are still very poorly understood, however, and depart from the dominant feedforward view that usually gives very little credit to horizontal interactions and intracortical processing.^92^^–^^96^ The VSDI signal is complicated and reflects the population membrane potential changes mostly influenced by sub- and not suprathreshold, spiking, activity. We believe that, in addition to the inherent technical difficulties of the method, both the unknown scale and the nonstandard origin of the signal keeps this tool from being used by a majority of researchers in neuroscience systems. However, there is no ideal technique and all existing approaches exhibit methodological drawbacks to be considered. To start with, the gold standard “single-unit activity” must be interpreted with caution since spike identification is never perfect and can be contaminated by neighboring cells, as shown by the literature on spike sorting techniques.^97^^–^^99^ At the other end of the recording spectrum, the origin and the link of fMRI signal to neuronal response still remains unclear, even after huge efforts to better understand it through both models and experiment.^100^^,^^101^ Similarly, the inverse problem for EEG recordings is still far from being solved despite a lot of computational efforts.^102^ Closer to VSDI, two-photon mostly records calcium fluorescence, whose link to spiking activity is nontrivial and needs the development of sophisticated algorithms.^103^^,^^104^ In all these techniques, computational studies are essential to their development and appropriate use. In this way, the development of VSDI similarly depends on these approaches to fill the gap of our current knowledge on mesoscopic neuronal operations.

On one hand, computational models are, therefore, very important to overcome these hurdles by providing a solid framework to better understand the types of operations subtending the signal’s dynamics and their neuronal origins. Through the close interaction between models and physiology, VSDI data can demonstrate that such operations cannot be summarized in terms of a simple excitatory-inhibitory balance of the feedforward drive.^105^ From this perspective, the fact that the signal has a subthreshold origin can be seen as an advantage, since it allows probing the network operations occurring at population level by highlighting their synaptic origin and processing.^106^^–^^111^ To bridge this gap between network synaptic operations and the mesoscopic population response, computational models are crucial. More generally, theoretical considerations about the information processing and encoding capacities that reside specifically at the mesoscopic scale are clearly needed, both for improving our understanding of the experimental results, but also to generate tractable predictions. Advances in signal processing, on the other hand, are essential to generate standardized and comparable datasets among studies. It is also necessary to remove artifacts that could potentially contaminate the signal and hinder access to analysis of single trials. Since the signal is spatio-temporally inseparable (i.e., cannot be described by two independent spatial and temporal functions), because of inherent propagations due to feedforward and feedback divergence, but also to intracortical connectivity, analyzing data at the single-trial level is essential to understand its dynamics, which are easily corrupted by averaging.^86^^,^^112^^,^^113^ Here, computational approaches can help to describe theoretically the type of spatio-temporal dynamics occurring, from single cycle propagating wave, to standing and traveling waves.^114^

Finally, the inherent methodological difficulties linked to this optical imaging technique have also hindered its development. It is clear that methodological advances such as genetically encoded voltage indicators^115^^–^^117^ can profoundly help to popularize the technique. However, the theoretical weaknesses linked to our poor understanding of mesoscopic neuronal operations will remain if the computational community does not become more strongly involved. In this review, we have discussed several recent efforts for developing computational methods that have improved our knowledge and understanding of the VSDI signal. However, we believe that there is still a large gap in terms of conceptual framework to interpret and guide the experimental results obtained at this scale. This will only be possible through a closer interactions and collaboration between the physiological and computational communities. Only then, the direction initiated by the remarkable work of Amiram Grinvald will continue to pave the way for understanding the mysteries still hidden at the mesoscopic scale.
